# Hepatitis E Virus Genotype 4 in Yak, Northwestern China

**DOI:** 10.3201/eid2012.131599

**Published:** 2014-12

**Authors:** Fang Xu, Yangyang Pan, Abdul Rasheed Baloch, Lili Tian, Meng Wang, Wang Na, Lingqiang Ding, Qiaoying Zeng

**Affiliations:** Gansu Agricultural University, Lanzhou, China (F. Xu, Y. Pan, W. Na, Q. Zeng);; Northwest A&F University, Yangling, China (A.R. Baloch, M. Wang);; China Animal Health and Epidemiology Center, Qingdao, China (L. Tian);; Sichuan Agricultural University, Yaan, China (L. Ding)

**Keywords:** hepatitis E, hepatitis E virus, yak, China, hepatitis, viruses, genotype 4, Bos grunniens

**To the Editor:** Hepatitis E virus (HEV; family *Hepeviridae*, genus *Hepevirus*) is a positive-stranded RNA virus with a genome of ≈7.2 kb that contains 3 open reading frames (*1*,*2*). On the basis of sequence analysis, mammalian HEVs are classified into 4 recognized genotypes (*3*,*4*). HEV genotypes 1 and 2 are restricted to humans and are often associated with large outbreaks and epidemics in developing countries, especially in Africa and Asia. Genotypes 3 and 4 are zoonotic and have been detected in humans, pigs, and other animal species (*1*,*3*–*6*). 

Yaks (*Bos grunniens*) live on the cold highland (altitude >3,000 m, average annual temperature <0°C) surrounding the Qinghai-Tibet Plateau, which includes Qinghai and Gansu Provinces in northwest China. Domestic yaks are usually slaughtered for meat at 3 years of age. Infectious pathogens in yaks have been reported only recently (*7*,*8*). On the basis of the high prevalence of HEV in human and pigs in China and close human–yak contact in the Tibet region (*4*,*5*), we sought to determine if HEV infects yaks.

During March–September 2013, we collected 167 fecal samples from yaks <3 years of age; 92 were from Qinghai Province (56 <1 year of age) and 75 from Gansu Province (48 <1 year of age). Soon after sampling, 10% (wt/vol) fecal suspensions were prepared by using sterile phosphate-buffered saline (0.01 mmol/L phosphate, pH 7.2–7.4; 0.15 mmol/L NaCl, 0.1% diethyl pyrocarbonate). After centrifugation, supernatants were separated, and total RNAs were extracted by using TRIzol reagent (Invitrogen, Carlsbad, CA, USA). RNAs were used as templates to amplify full-length cDNA by reverse transcription PCR (RT-PCR; SuperScript III Synthesis Kit, Invitrogen), according to the manufacturer’s instructions. A positive control sample (GenBank accession no. JU119961) and negative control (water) were included. 

Briefly, 10 pairs of primers were designed based on HEV genotype 4 (GenBank accession nos. JU119961, JQ740781, AB291965, and AB602440) (Technical Appendix Table 1) to obtain the HEV genome consisting of all the 3 open reading frames. RT-PCR was then performed in a 25-μL volume containing 2-μL templates and 0.1 μmol/L of each primer; cycles were 94°C for 2 min, followed by 38 cycles of 94°C for 30 s, 59°C for 30 s, and 72°C for 1 min, with a final extension step of 10 min at 72°C. RT-PCR–amplified DNA fragments of the expected sizes were sequenced in a 310 Genetic Analyzer/Sanger Sequencer (Invitrogen). The complete genome sequences were assembled on the basis of 10 amplified sequences. Phylogenetic analysis was performed for the complete genome sequences of the detected sequences compared with all other mammalian HEV sequences available in GenBank.

We found that 3 (3.26%) of the 92 samples were positive for the HEV genotype 4 genome sequence; all samples were from yaks <1 year of age from Qinghai Province (Technical Appendix Table 2). Sequence analysis revealed 100% identity of the full-length genomes of the 3 HEV sequences (7,234 bp; sequence submitted to GenBank as CHN-QH-YAK, accession no. KF736234). Phylogenetic analysis demonstrated that all 19 mammalian HEVs grouped into 4 clades corresponding to the 4 HEV genotypes. The sequence we identified belonged to genotype 4 and was most closely related to China/Xinjiang/swine (GenBank accession no. JU119961; 99.14% identity) and to China/Nanjing/human (GenBank accession no. JQ740781; 93.84% identity) (Figure).

**Figure:**
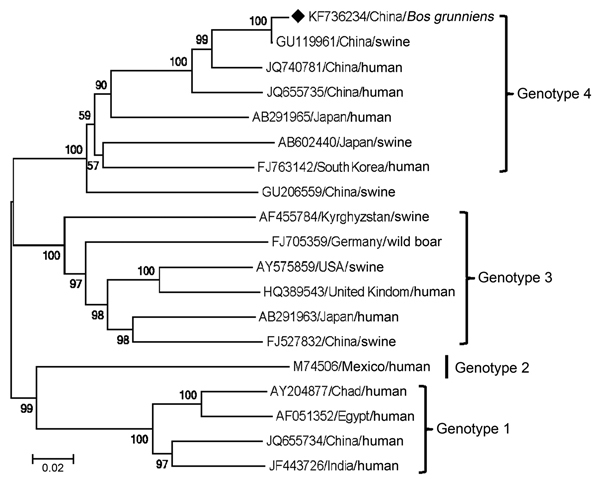
Phylogenetic analysis of hepatitis E virus (HEV) based on the complete genome sequences of HEVs using the neighbor-joining method with MEGA 4.0 software (http://www.megasoftware.net). Black diamond indicates the newly identified yak HEV sequence from Qinghai, China (GenBank accession no. KF736234). Another 18 sequences were collected from GenBank, including 7 sequences of genotype 4 (GU119961, JQ740781, JQ655735, AB291965, AB602440, FJ763141, GU206559), 6 of genotype 3 (AF455784, FJ705359, AY575859, HQ389543, AB291963, FJ527832), 1 of genotype 2 (M74596), and 4 of genotype 1 (AY204877, AF051352, JQ655734, JF443726). Bootstrap values of >50% are indicated for the corresponding nodes based on a bootstrapping with 1,000 replicates. GenBank accession numbers and geographic and animal species origin are shown. Scale bar indicates nucleotide substitutions per site.

We found HEV genotype 4 infection in yaks, but prevalence was low (3.26%), and only young yaks in Qinghai Province were affected. In comparison, studies have shown that swine are an established reservoir of HEV worldwide (*3*,*5*,*9*) and that 20%–100% of pigs are infected with HEV (*3*,*4*,*9*,*10*). Our findings suggest that yak is an emerging but imperfect host for this virus. The HEV genome sequence derived from infected yak shared 99.14% identity with the China/Xinjiang/swine isolate, suggesting the Qinghai isolate evolved and was transmitted from the Xinjiang swine isolate. Like the swine isolate, this yak isolate probably possesses the potential to infect humans. Because persons in the Tibet region eat undercooked yak milk and meat, yaks may become an emerging reservoir of HEV genotype 4. 

In addition, the high sequence identity (93.84%–99.14%) among isolates from China, including China/Qinghai/yak, China/Xinjiang/swine, China/Nanjing/human, and China/Beijing/human, demonstrates a complicated, transregional and cross-species transmission cycle for HEV genotype 4 in China. We cannot determine why yaks in Qinghai Province were affected and those in Gansu Province were not, but a neighboring pig farm may have served as a source for HEV transmission. More research is needed to determine the prevalence of HEV genotype 4 in various human and animal populations with concomitant virus isolation and phylogenetic analysis.

Technical Appendix Frequency of hepatitis E virus (HEV) detected by RT-PCR assay in fecal samples from domestic yaks of different ages in 2 provinces of northwest China and sequences of primers for cloned HEV genome sequence that contained all open reading frames.
