# Supplementary material for: Hepatitis E Virus Genotype 4 in Yak, Northwestern China
Source: Emerg Infect Dis. 2014 Dec;20(12):2182–4. doi: 10.3201/eid2012.131599 (PMC4257818; doi:10.3201/eid2012.131599)
Supplement: Technical Appendix — Frequency of hepatitis E virus (HEV) detected by RT-PCR assay in fecal samples from domestic yaks of different ages in 2 provinces of northwest China and sequences of primers for cloned HEV genome sequence that contained all open reading frames. [file 13-1599-Techapp-s1.pdf]

DOI: http://dx.doi.org/10.3201/eid2012.131599

# Hepatitis E Virus Genotype 4 in Yak, Northwestern China

## Technical Appendix

Technical Appendix Table 1. Frequency of hepatitis E virus detected by RT-PCR assay in fecal samples from domestic yaks of different ages in 2 provinces of northwest China

| Province | Animal age, mo | No. (%) samples tested | No. (%) positive |
|---|---|---|---|
| Qinghai | ≤12 | 56 (60.87) | 3 (5.36) |
| | 12–36 | 36 (39.13) | 0 |
| Gansu | ≤12 | 48 (64.00) | 0 |
| | 12–36 | 27 (36.00) | 0 |

Technical Appendix Table 2. Sequences of primers for cloned hepatitis E virus genome sequence contained all open reading frames

| Primer | Sequence, 5′ → 3′ | Site (nt)* |
|---|---|---|
| P1 | F-CACGTATGTGGTCGACGCC | 8–26 |
| | R-GAAGAGCATAAGCCTGTCCCA | 984–1004 |
| P2 | F-CTGCCTGCTCTACTAAATCCAYAT | 937–960 |
| | R-CGATTGAACCTRTACAAGGCTGA | 1996–1974 |
| P3 | F-TGGCTGCYGGYCCACATAGTC | 1828–1848 |
| | R-CGTGCCGTGTCCTCAGTAATA | 2867–2887 |
| P4 | F-ATCTTACTGAGCCAGCCATAGC | 2806–2827 |
| | R-ACGAGACACATCACGGCTRCAC | 4178–4199 |
| P5 | F-ACCTAGYCAGCGTAAAGCTGTC | 3962–3983 |
| | R-CCACATTCGTTAACTTTCGCA | 4962–4982 |
| P6 | F-GACCCTCCCTGATGTTGTTAGG | 4859–4880 |
| | R-CAATAGTGGACGTAAGCGGAGA | 5609–5630 |
| P7 | F-CCGCTTACGTCCACTATTGCTA | 5612–5633 |
| | R-AGTGCACCAGTATAAGGCGTATTG | 6055–6078 |
| P8 | F-CCTAGTGAGCGCCTGCACTAT | 5027–5947 |
| | R-CGAGAATAGAACAATTGTCCACCA | 6361–6384 |
| P9 | F-AGYTACCCGCTTTATGAAAGYTCT | 6223–6246 |
| | R-TGAAATRCAGACRCGGTGRCC | 6914–6934 |
| P10 | F-TGCTAATGACGTGCTCTGGCT | 6586–6606 |
| | R-TGCGTTCCGCGCTCCCTGA | 7149–7241 |

*Site on the genome of China swine strain (GenBank accession no. GU119961). R = A/G; Y = C/T.
